# Measurement of proptosis using computed tomography based three-dimensional reconstruction software in patients with Graves’ orbitopathy

**DOI:** 10.1038/s41598-020-71098-4

**Published:** 2020-09-03

**Authors:** Jung Huh, Sang Joon Park, Jeong Kyu Lee

**Affiliations:** 1 Department of Ophthalmology, Chung-Ang University College of Medicine, Chung-Ang University Hospital, 102 Heukseok-ro, Dongjak-gu, Seoul, 06973 Korea; 2 Department of Radiology, Seoul National University Hospital, 101 Daehak-ro, Jongno-gu, Seoul, 03080 Korea

**Keywords:** Biomarkers, Diseases, Medical research

## Abstract

The evaluation of proptosis is essential for the diagnosis of orbital disease. We have developed a computed tomography (CT)-based three-dimensional (3D) reconstruction software to measure the degree of proptosis. To verify clinical usefulness and reliability, the degree of proptosis was measured in 126 patients with Graves’ orbitopathy (GO) with 3D reconstruction software and compared with those obtained with Hertel exophthalmometer and CT. The proptosis values measured by 3D reconstruction software, CT, and Hertel exophthalmometer were closely related to each other, but showed significant differences (*p* < 0.001). In contrast, the amount of change in proptosis after orbital decompression were not different among the three measurements (*p* = 0.153). The intra-observer correlation coefficients of the 3D reconstruction software, CT, and Hertel exophthalmometer measurements were 0.997, 0.942, and 0.953, respectively. In patients with strabismus, the intra-observer correlation coefficient of CT and Hertel exophthalmometer decreased to 0.895 and 0.920, respectively, but the intra-observer correlation coefficient of the 3D reconstruction software did not change to 0.996. The inter-observer correlation coefficients of CT and 3D reconstruction software for three different ophthalmologists were 0.742 and 0.846, respectively. In conclusion, the measurement of proptosis by 3D reconstruction software seems to be a reliable method, especially in the presence of eyeball deviation.

## Introduction

Measurement of ocular proptosis is essential for the diagnosis of orbital diseases such as Graves’ orbitopathy (GO), orbital tumor, and orbital fracture. There are various types of devices available for measuring the degree of proptosis. The Hertel exophthalmometer, invented by Hertel in 1905, is the most widely used device to date^1,2^. It estimates the degree of proptosis from the lateral orbital rim to the corneal surface, perpendicular to the frontal plane^3^; however, the Hertel exophthalmometer has been criticized for its low reliability and accuracy^4^. Musch et al.^5^ showed a statistically significant inter-observer difference with the Hertel exophthalmometer, with a 61–80% agreement. Furthermore, differences in proptosis measurements have been reported between Hertel exophthalmometers made by different companies^1,6,7^. The differences in readings may result from misplacement of the foot plates, strabismus, asymmetry of the lateral orbital rims, compression of soft tissues, parallax errors, or the lack of a uniform measurement technique^8,9^.

To compensate for these limitations, computed tomography (CT) has been used to measure proptosis and is reported to produce more accurate data^10–14^; however, proptosis measurement using CT is also associated with some limitations. Since the apex of the cornea and interzygomatic line cannot be included in the same plane on a two-dimensional (2D) CT scan, the level of the measured CT slice may not correspond to the area of maximal proptosis. In addition, it can cause errors in the process of manually specifying the point of interest and measuring the distance. Lastly, the measured distance is limited to only a 2D space. Errors in measurements can result from an eyeball position that is not centered by aligning the mid-sagittal line perpendicular to the straight line, which is typical in patients with vertical strabismus.

CT-based three-dimensional (3D) reconstruction software was introduced to overcome these limitations. 3D reconstruction software utilizes the CT slice of the entire eyeball and the interzygomatic plane to measure the grade of proptosis semi-automatically in 3D space. Measurements using 3D reconstruction software are expected to produce more accurate values.

The purpose of this study was to introduce a novel proptosis measurement method and to evaluate reliability in the measurement of exophthalmos using 3D reconstruction software.

## Results

A total of 126 subjects were included in this study. The average age was 38.2 ± 13.5 years (with a range of 15–79 years); there were 39 (31.0%) male patients and 87 (69.0%) female patients. There were 21 (16.7%) patients with strabismus and 20 (15.9%) patients who underwent orbital decompression surgery (Table 1).

**Table 1:** Characteristics of the study population.

	Study population (N = 126)
Sex (Male / Female)	39 / 87
Age (years)	38.2 ± 13.5 (15–79)
Patients with strabismus (exotropia/esotropia/hypertropia)	21 (6/5/10)
Underwent orbital decompression	20

**Figure 1:**
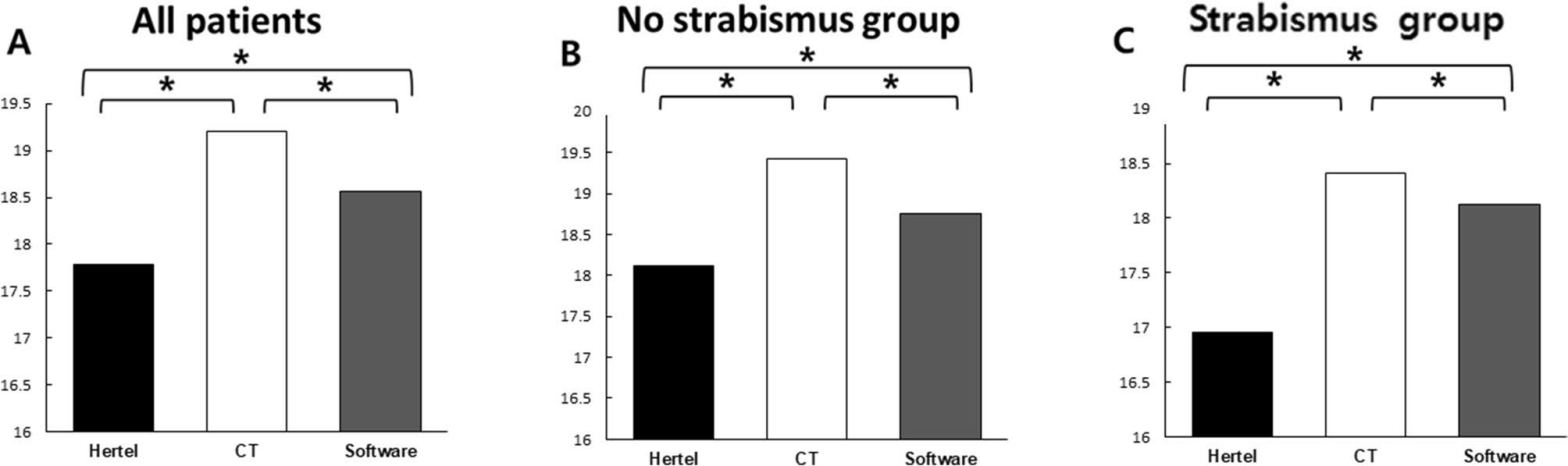
Comparison of the Hertel exophthalmometer, computed tomography (CT), 3D reconstruction software. There were significant differences in measurements among three measurements in Graves orbitopathy patients with and without strabismus group. (**A**) Proptosis measurement in All patients (*: Bonferroni correction, Hertel vs. CT, CT vs. 3D reconstruction software, Hertel vs. 3D reconstruction software : *p* < 0.001) (**B**) Proptosis measurement in no strabismus group (*: Bonferroni correction, Hertel vs. CT, CT vs. 3D reconstruction software, Hertel vs. 3D reconstruction software : *p* < 0.001) (**C**) Proptosis measurement in strabismus group (*: Bonferroni correction, Hertel vs. CT : *p* = 0.020, CT vs. 3D reconstruction software : *p* = 0.041, Hertel vs. 3D reconstruction software : *p* = 0.024).

Table 2 shows values of proptosis as measured by Hertel exophthalmometer, CT scan, and 3D reconstruction software. There was a statistically significant difference among the mean proptosis values measured by these methods (*p* < 0.001). In subgroup analysis, the values of proptosis measured for non-strabismus and strabismus patients showed significant differences between the three measurements (*p* < 0.001, 0.008) (Fig. 1).

**Table 2:** Comparison of the Hertel exophthalmometer, computed tomography (CT), and 3D reconstruction software.

	All patients (mm)	Non-strabismus group (mm)	Strabismus group (mm)
Hertel	17.78 ± 3.20	18.12 ± 3.26	16.95 ± 3.31
CT	19.21 ± 3.13	19.43 ± 3.20	18.41 ± 3.72
Software	18.56 ± 3.29	18.75 ± 3.39	18.12 ± 3.99
*p* value*	< 0.001^‡^	< 0.001^§^	0.008^||^

*CT*  computed tomography.

*Repeated measures one-way analysis of variance, ^‡^Bonferroni correction, Hertel vs. CT, CT vs. 3D reconstruction software, Hertel vs. 3D reconstruction software: *p* < 0.001, ^§^Bonferroni correction, Hertel vs. CT, CT vs. 3D reconstruction software, Hertel vs. 3D reconstruction software: *p* < 0.001, ^||^Bonferroni correction, Hertel vs. CT: *p* = 0.020, CT vs. 3D reconstruction software: *p* = 0.041, Hertel vs. 3D reconstruction software : *p* = 0.024.

Proptosis measures for patients who underwent surgery showed significant differences among the three measurements before and after surgery (*p* = 0.024, *p* = 0.012) (Table 3). The difference between preoperative and postoperative proptosis of the three measurements was statistically significant (*p* < 0.001). No other changes in measured proptosis after surgery were significantly different among the three measurements (*p* = 0.153).

**Table 3:** Comparison of the Hertel exophthalmometer, computed tomography (CT), and 3D reconstruction software in patients who underwent orbital decompression.

	Preoperative (mm)	Postoperative (mm)	Difference (mm)	*p* value^†^
Hertel	18.96 ± 2.42	15.70 ± 1.72	3.26 ± 1.83	< 0.001
CT	20.12 ± 2.94	17.46 ± 2.42	2.93 ± 2.12	< 0.001
Software	19.74 ± 2.79	16.62 ± 2.52	3.12 ± 2.05	< 0.001
*p* value*	0.024	0.012	0.153	

*CT*  computed tomography.

*Repeated measures one-way analysis of variance, ^†^Paired t test between preoperative and postoperative values.

Table 4 shows the comparison of Hertel exophthalmometer, CT scan, and 3D reconstruction software according to the degree of proptosis. In the patients with proptosis of less than 21 mm, there was a significant difference in the measurements between the three methods; the highest value was obtained by CT followed by the 3D reconstruction software and Hertel exophthalmometer (*p* < 0.001). In patients with proptosis of more than 21 mm, the CT measurement was significantly higher than other measurements, whereas there was no significant difference between the value measured by the 3D reconstruction software and Hertel exophthalmometer. (*p* = 0.618).

**Table 4:** Comparison of the Hertel exophthalmometer, computed tomography (CT), and 3D reconstruction software according to the degree of proptosis.

	< 21 mm proptosis (187 eyes)	> = 21 mm proptosis (65 eyes)
Hertel	16.43 ± 2.27	21.88 ± 1.54
CT	17.95 ± 2.46	22.83 ± 1.77
Software	17.20 ± 2.46	22.14 ± 1.87
*p* value*	< 0.001^‡^	0.002^§^

*CT* computed tomography.

*Repeated measures one-way analysis of variance, ^‡^: Bonferroni correction, Hertel vs. CT: *p* < 0.001, CT vs. 3D reconstruction software: *p* = 0.008, Hertel vs. 3D reconstruction software : *p* = 0.003, ^§^Bonferroni correction, Hertel vs. CT: *p* = 0.002, CT vs. 3D reconstruction software: *p* = 0.037, Hertel vs. 3D reconstruction software : *p* = 0.618.

The intra-observer correlation coefficients (Cronbach alpha) were 0.953 (Hertel), 0.942 (CT), and 0.997 (3D reconstruction software) for the total patient population (Table 5). In non-strabismus patients, the intra-observer correlation coefficients were 0.933 (Hertel), 0.921 (CT), and 0.997 (3D reconstruction software). In strabismus patients, the intra-observer correlation coefficients were 0.920 (Hertel), 0.895 (CT), and 0.996 (3D reconstruction software). The interclass correlation coefficients (ICCs) of CT and 3D reconstruction software measurements among the three ophthalmologists were 0.742 and 0.846, respectively; these values are significantly different (*p* < 0.05).

**Table 5:** Intra-observer reliability (Cronbach’s alpha) of the Hertel exophthalmometer, computed tomography (CT), and 3D reconstruction software.

	All patients	Non-strabismus group	Strabismus group
Hertel	0.953	0.933	0.920
CT	0.942	0.921	0.895
Software	0.997	0.997	0.996

*CT* computed tomography.

**Figure 2:**
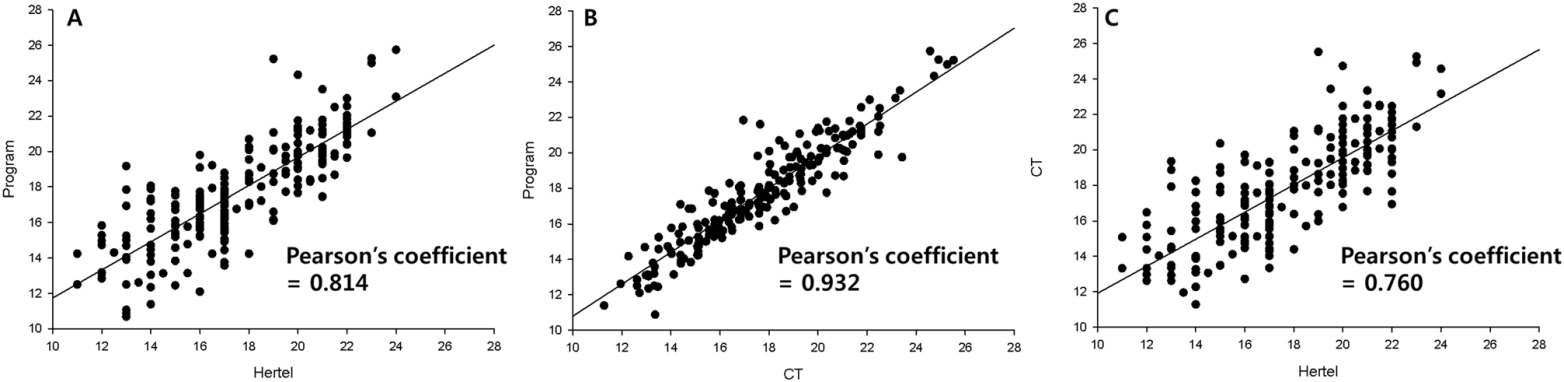
Correlations between Hertel exophthalmometer, computed tomography (CT), and 3D reconstruction software-assisted measurements. (**A**) Hertel and 3D reconstruction software (Pearson’s correlation coefficient = 0.814) (**B**) CT and 3D reconstruction software (Pearson’s correlation coefficient = 0.932), (**C**) Hertel and CT (Pearson’s correlation coefficient = 0.760).

**Figure 3:**
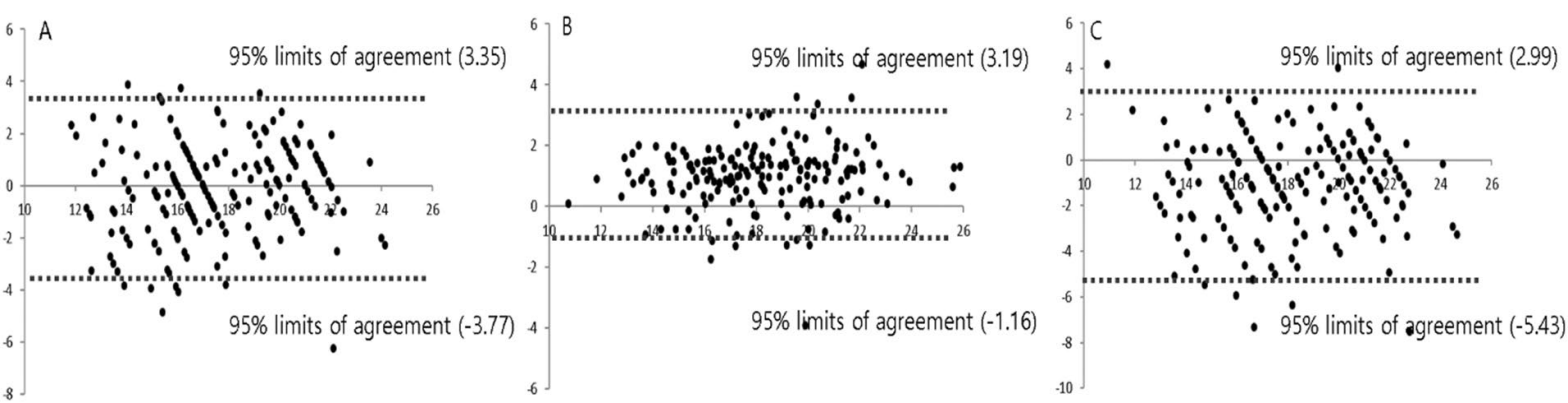
Bland–Altman plots comparing the Hertel exophthalmometer, computed tomography (CT), and 3D reconstruction software. (**A**) Hertel and 3D reconstruction software, (**B**) CT and 3D reconstruction software, and (**C**) Hertel and CT.

Strong correlations between measurements were observed; the Pearson’s correlation coefficient was 0.932 (CT and 3D reconstruction software), 0.814 (Hertel and 3D reconstruction software), and 0.760 (Hertel and CT) (Fig. 2). Weaker correlations between measurements in the strabismus group were observed: the Pearson’s correlation coefficient was 0.853 (CT and 3D reconstruction software), 0.765 (Hertel and 3D reconstruction software), and 0.713 (Hertel and CT). On the Bland–Altman plot, the 95% limits of agreement (LOAs) were -3.77 to 3.35 mm (Hertel and 3D reconstruction software), -1.16 to 3.19 mm (CT and 3D reconstruction software), and -5.43 to 2.99 mm (Hertel and CT) (Fig. 3) The LOA between Hertel and 3D reconstruction software was smaller than that between Hertel and CT.

## Discussion

We aimed to determine whether proptosis measurements using 3D reconstruction software are more reliable and useful compared to proptosis measurements using the Hertel exophthalmometer and CT. There were significant differences in measurements among the Hertel exophthalmometer, CT, and the 3D reconstruction software in GO patients with and without strabismus. The mean value of the 3D reconstruction software measurement was longer than that of the Hertel exophthalmometer by 0.78 mm and shorter than that of CT by 0.65 mm. Though the difference between the proptosis values measured by the Hertel exophthalmometer and those with the 3D reconstruction software in patients with more than 21 mm proptosis was smaller, the CT measurements were still 0.98 mm larger than Hertel measurements. Kim et al. reported that the value of protrusion measured with CT was longer than that of the Hertel exophthalmometer by 0.3–1.4 mm in a Korean population^4^. This difference could be because of the difference in patient position during examination: supine for CT and 3D reconstruction software and sitting for the Hertel exophthalmometer. In addition, the reference points such as the interzygomatic line and the plane are located at the rear rather than at the foot plates of the Hertel exophthalmometer owing to the presence of skin and soft tissue. Otherwise, there were no significant differences in proptosis changes after surgery among these three measurement methods. This suggests that these measurements should not be used interchangeably, but instead be considered as relative values. Changes in the 3D reconstruction software-assisted measurements could be used in the evaluation of surgical outcomes as accurate data.

The intra-observer correlation coefficients of the 3D reconstruction software, CT, and Hertel exophthalmometer measurements were 0.997, 0.942, and 0.953, respectively. In strabismus patients, the intra-observer correlation coefficients for the 3D reconstruction software, CT, and Hertel exophthalmometer were 0.996, 0.895, and 0.920, respectively. The ICCs for CT and 3D reconstruction software between three different ophthalmologists were 0.742 and 0.846, respectively. This ICC for 3D reconstruction software is interpreted as excellent agreement. Our study was limited by the fact that we did not evaluate the inter-observer variability of the Hertel exophthalmometer; however, a high variability in the Hertel exophthalmometer measurements between different observers has been widely reported, from 30 to 80%^3,5^. Considering these results, 3D reconstruction software is the most reliable of the tested methods and would be useful when there is a large difference between the Hertel exophthalmometer and CT measurements, such as strabismus, or if other test are less reliable due to inconsistent measurements.

Errors in measurement using the Hertel exophthalmometer occur when the foot plate is incorrectly placed in the lateral orbital rim, and there can also be interpersonal differences in estimating the same patient^15^. Measurement requires an intact lateral orbital rim for fixation, which may be asymmetrical due to trauma or surgery^16^. Another factor that influences the results is that the base value may be different according to the state of the tissue and the degree of compression. If there is swelling around the lateral orbital margin due to dysthyroid ophthalmopathy or other disease, it greatly enhances the error. The CT approach is also associated with some limitations. As only one slice of the CT is used for measurement, the level of the CT slice may not correspond to the area of maximal proptosis. In addition, as the interzygomatic line and perpendicular line are drawn by the user, there can be differences in estimation for the same patient. The semi-automated measurements of the 3D reconstruction software may reduce observer bias resulting from subjective human evaluation.

Eyeball deviation in patients with GO is mainly accompanied by severe EOM limitation. In patients with strabismus and EOM limitation, the displaced array of the eyeball could change the frontal plane of the skull and the relative position of the eyeball when measured using the Hertel exophthalmometer. The limitation of EOM can interfere with central fixation of the deviated eye, making it more difficult to accurately measure the degree of proptosis with the Hertel measurement. The displaced array of the eyeball also challenges the selection of an axial section and the drawing of the perpendicular line for measurement using CT. In the clinical setting, up to 1 mm differences in exophthalmometer readings are considered acceptable^17^. However, the difference between the measurements with CT and the Hertel exophthalmometer was 1.46 mm in the patients with strabismus, and the difference in degree of proptosis by more than 1 mm in both eyes can sometimes significantly affect the patient’s surgical satisfaction. In this situation, it would be difficult to trust both measurements, and 3D reconstruction software may be a good alternative. Because measurements using 3D reconstruction software use the whole section of CT and automatically draw a perpendicular line in 3D space, these shortcomings of the other methods can be resolved.

Measurements using 3D reconstruction software were strongly correlated with those of the Hertel exophthalmometer and CT, as indicated by Pearson correlation coefficients of 0.814 and 0.932, respectively. 3D reconstruction software measurements were more closely related to CT measurements because the 3D reconstruction software is based on CT data. Weaker correlations between measurements in the strabismus group suggest that the displaced array of the eyeball affects measurement. The 95% LOAs between the Hertel exophthalmometer and 3D reconstruction software were smaller than those between the Hertel and CT. Measurements using 3D reconstruction software are more useful than those from CT when used with the Hertel exophthalmometer. The wider LOAs between different measurements should be considered carefully. The readings of these tools are not interchangeable as they are not equivalent, especially in the presence of strabismus.

In conclusion, measurements using the Hertel exophthalmometer, CT, and 3D reconstruction software were strongly correlated but showed significant differences. As measurements using 3D reconstruction software showed a higher reliability than those of the Hertel exophthalmometer and CT, this appears to be the most reliable method for measuring ocular protrusion.

## Methods

The study was approved by the institutional review board (IRB) committee of Chung-Ang University Hospital in Seoul, South Korea, and the requirement for informed consent was waived by the IRB of Chung-Ang University Hospital. Image acquisition, processing, and analysis were performed according to the tenets of the Declaration of Helsinki.

### Study subject

A total of 126 patients diagnosed with GO who were seen at the clinic were recruited. Patients with a history of trauma, pregnant patients, those with an incomplete set of CT images, and those with rapid progression of proptosis and symptoms were excluded. Patients who underwent lateral orbital wall decompression were also excluded from the study. Among the subjects, those with more than 15 prism diopters of vertical or horizontal eyeball deviation were subdivided into strabismus group.

All patients underwent comprehensive ophthalmologic examinations and CT scan. Clinical records including age, gender, previous history, type and degree of strabismus, and degree of proptosis were collected for review. Measurement of proptosis by the Hertel exophthalmometer and CT scan was taken within a week. Patients who underwent orbital decompression were evaluated for grade of proptosis and underwent CT scanning 3 months post-operatively. Orbital decompression was performed with medial and inferior wall decompression. To evaluate intra- and inter-observer reliability, measurements using CT and 3D reconstruction software were repeated three times by the same examiner and performed by three different examiners who were blinded to the previous measurements.

### Proptosis measurement by Hertel exophthalmometer

Degree of proptosis was independently measured by one experienced clinical observer (ophthalmologist) who used the Hertel exophthalmometer (Oculus Inc., Arlington, VA, USA). Patients were re-examined by the same observer within 1 month. Measurements were taken with the patient’s head in the primary position and the examiner’s eye at the same level as the patient’s eyes in a well-lit room. The measurement was the distance between the point on the temporal orbital rim at the deepest palpable point of the angle and the apex of the cornea. Right and left eye readings were performed sequentially without removing the instrument from the orbital rims. The measurements were recorded to the nearest 0.5 mm^18^.

### Proptosis measurement by CT scan

All patients were scanned with a 256-slice MDCT scanner (Brilliance 256; Philips Medical Systems, OH, USA), as previously described^19^. Orbital CT scans were obtained using contiguous axial slices, with the patient’s head positioned parallel to the Frankfurt plane. Patients were asked to look at a fixed point, and the scanning parameters were as follows: 120 kV, 150 mAs, 64 × 0.625 mm detector configuration, 1 mm slice thickness, and 1 mm slice increment. Measurement of proptosis was performed on the CT image by drawing a horizontal line between the lateral orbital rims on an axial plane that bisects the lens and then drawing a perpendicular line forward to the posterior surface of the cornea^18^. The posterior surface of the cornea was chosen because it can be difficult to define the anterior surface of the cornea on CT.

### Proptosis measurement by 3D reconstruction software

The CT data were digitally transferred from the PACS workstation to a personal computer, and the 3D reconstruction software imported the axial sections of the CT images. Then, our PC-based 3D reconstruction software was used for eyeball segmentation and fully automated quantification of computerized features, implemented with a dedicated C +  + language with MFC (Microsoft Foundation Classes, Microsoft, Redmond, WA)^20–25^. The overall procedure of this analysis scheme comprised 4 major stages as follows (Supplementary Video S1 for the procedure). First, eyeball segmentation was conducted semi-automatically. Then, a 3D interzygomatic plane was composed with 3 user-defined reference points. After finishing these steps, a normal perpendicular vector from the 3D interzygomatic plane to the end of the eyeball surface was calculated. Finally, the length of proptosis based on 3D CT images was computed quantitatively.

**Figure 4:**
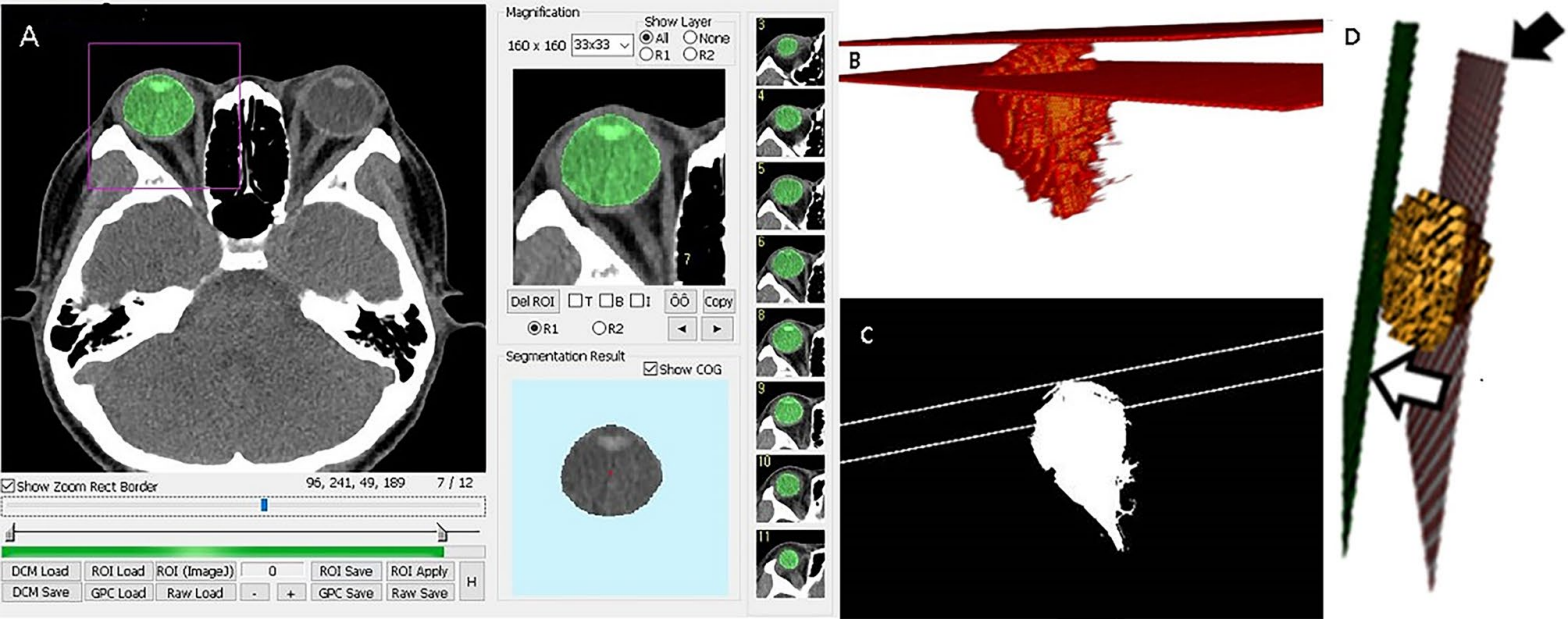
Example images were produced using our 3D reconstruction software. (**A**) Recognizing the eyeball of the whole axial Section (60–70 section) of computed tomography using automatic segmentation. (**B**) 3D rendering images of the interzygomatic plane and the plane of eyeball surface from a normal vector on the segmented eyeball (**C**) Binary image for mathematical verification (**D**) 3D reconstruction of the eyeball (yellow sphere) and the interzygomatic plane (black arrow). A perpendicular line (white arrow) drawn from the recognized eyeball surface to the interzygomatic plane by 3D reconstruction software.

Semi-automatic segmentation of the eyeball based on a graph-cut technique was performed by an observer^26^. The foreground seed for the targeted eyeball area and background seeds for the non-eyeball region were delineated in one arbitrary CT slice to recognize the accurate eyeball area of entire axial CT sections (Fig. 4A). Then, segmentation was performed automatically based on the intensity map model using global optimization of a cost function. Due to the semi-automatic recognition algorithm, it took less than 10 min for one user to assess the whole axial section. Next, to compose the 3D interzygomatic plane, the user placed three reference points: two along the anterior border of the bilateral orbital rim on an axial section in which the lens was most largely seen and one along the anterior border of the ipsilateral orbital rim on an axial section in which the second largest lens was seen. After that, the software calculated the center of mass at the junction of the eyeball and the interzygomatic plane. From the center of mass on the 3D interzygomatic plane, a normal vector was cast perpendicular to the end of the recognized eyeball surface (Fig. 4B,C). Finally, the length of a perpendicular line that represents the degree of proptosis based on 3D CT images was computed quantitatively (Fig. 4D).

### Statistical analysis

Data were expressed as the mean ± SD. Repeated measures one-way analysis of variance (RM-ANOVA) with Bonferroni correction was used to compare proptosis measurements by different modalities. To measure the strength of the linear association between modalities, correlations between the modalities were analyzed by Pearson correlation. The Bland–Altman method was used to analyze the agreement between the modalities. Intra-observer correlations of modality were calculated using Cronbach alpha, as was the intraclass correlation coefficient, to assess the level of agreement for each measurement. To determine inter-observer reliability, measured values were evaluated using interclass correlation coefficients (ICCs). Subgroup analysis was performed for patients with strabismus and patients who underwent surgery. Statistical analysis was performed using SPSS 20.0 (SPSS Inc., Chicago, IL, USA). Statistical significance was defined as *p* < 0.05.

## Supplementary information

Supplementary Video S1.

## References

[1] Dunsky IL (1992). Normative data for hertel exophthalmometry in a normal adult black population. Optom. Vis. Sci. Off. Publ. Am. Acad. Optom..

[2] Migliori ME, Gladstone GJ (1984). Determination of the normal range of exophthalmometric values for black and white adults. Am. J. Ophthalmol..

[3] O'Donnell NP, Virdi M, Kemp EG (1999). Hertel exophthalmometry: the most appropriate measuring technique. Br. J. Ophthalmol..

[4] Kim IT, Choi JB (2001). Normal range of exophthalmos values on orbit computerized tomography in Koreans. Ophthalmol. J. Int. d'ophtalmologie. Int. J. Ophthalmol. Zeitschrift fur Augenheilkunde.

[5] Musch DC, Frueh BR, Landis JR (1985). The reliability of Hertel exophthalmometry. Observer variation between physician and lay readers. Ophthalmology.

[6] Chang AA, Bank A, Francis IC, Kappagoda MB (1995). Clinical exophthalmometry: a comparative study of the Luedde and Hertel exophthalmometers. Aust. N. Z. J. Ophthalmol..

[7] Sleep TJ, Manners RM (2002). Interinstrument variability in Hertel-type exophthalmometers. Ophthalmic Plast. Reconstr. Surg..

[8] Frueh BR, Garber F, Grill R, Musch DC (1985). Positional effects on exophthalmometer readings in Graves' eye disease. Arch. Ophthalmol. (Chicago, Ill.: 1960).

[9] Ameri H, Fenton S (2004). Comparison of unilateral and simultaneous bilateral measurement of the globe position, using the Hertel exophthalmometer. Ophthalmic Plast. Reconstr. Surg..

[10] Hallin ES, Feldon SE (1988). Graves' ophthalmopathy: II. Correlation of clinical signs with measures derived from computed tomography. Br. J. Ophthalmol..

[11] Segni M, Bartley GB, Garrity JA, Bergstralh EJ, Gorman CA (2002). Comparability of proptosis measurements by different techniques. Am. J. Ophthalmol..

[12] Nkenke E (2003). Relative en- and exophthalmometry in zygomatic fractures comparing optical non-contact, non-ionizing 3D imaging to the Hertel instrument and computed tomography. J. Cranio-Maxillo-Fac. Surg. Off. Publ. Eur. Assoc. Cranio-Maxillo-Fac. Surg..

[13] Nkenke E (2004). Hertel exophthalmometry versus computed tomography and optical 3D imaging for the determination of the globe position in zygomatic fractures. Int. J. Oral Maxillofac. Surg..

[14] Ramli N, Kala S, Samsudin A, Rahmat K, Abidin ZZ (2015). Proptosis-correlation and agreement between hertel exophthalmometry and computed tomography. Orbit (Amsterdam, Netherlands).

[15] Kashkouli MB, Beigi B, Noorani MM, Nojoomi M (2003). Hertel exophthalmometry: reliability and interobserver variation. Orbit (Amsterdam, Netherlands).

[16] Jeon HB, Kang DH, Oh SA, Gu JH (2016). Comparative study of naugle and Hertel exophthalmometry in orbitozygomatic fracture. J. Craniofacial Surg..

[17] Lam AK, Lam CF, Leung WK, Hung PK (2009). Intra-observer and inter-observer variation of Hertel exophthalmometry. Ophthalmic Physiol. Opt. J. Br. Coll. Ophthalmic Opt. (Optometrists).

[18] Park NR, Moon JH, Lee JK (2019). Hertel exophthalmometer versus computed tomography scan in proptosis estimation in thyroid-associated orbitopathy. Clin. Ophthalmol..

[19] Byun JS, Moon NJ, Lee JK (2017). Quantitative analysis of orbital soft tissues on computed tomography to assess the activity of thyroid-associated orbitopathy. Graefes Arch. Clin. Exp. Ophthalmol..

[20] Ryu YJ (2014). Glioma: application of whole-tumor texture analysis of diffusion-weighted imaging for the evaluation of tumor heterogeneity. PLoS ONE.

[21] Song YS (2014). Volume and mass doubling times of persistent pulmonary subsolid nodules detected in patients without known malignancy. Radiology.

[22] Ahn SY (2015). Prognostic value of computed tomography texture features in non-small cell lung cancers treated with definitive concomitant chemoradiotherapy. Invest. Radiol..

[23] Hwang IP (2015). Persistent pure ground-glass nodules larger than 5 mm: differentiation of Invasive pulmonary adenocarcinomas from preinvasive lesions or minimally invasive adenocarcinomas using texture analysis. Invest. Radiol..

[24] Lee JH (2015). Value of computerized 3D shape analysis in differentiating encapsulated from invasive thymomas. PLoS ONE.

[25] Kim H (2016). Temporal changes of texture features extracted from pulmonary nodules on dynamic contrast-enhanced chest computed tomography: how influential is the scan delay?. Invest. Radiol..

[26] Boykov Y, Kolmogorov V (2004). An experimental comparison of min-cut/max-flow algorithms for energy minimization in vision. IEEE Trans. Pattern Anal. Mach. Intell..

